# Supplementary material for: An open-source framework for large-scale, flexible evaluation of biomedical text mining systems
Source: J Biomed Discov Collab. 2008 Jan 29;3:1. doi: 10.1186/1747-5333-3-1 (PMC2276192; doi:10.1186/1747-5333-3-1)
Supplement: Additional file 1 — Evaluation platform source code. The additional file contains the software evaluation framework discussed in this paper. The most current release can be downloaded from SourceForge.net [1]. See the accompanying README file for instructions on installation and use. Also included in the distribution is a collection of UIMA wrappers for some commonly used BioNLP tools and annotated corpora. [file 1747-5333-3-1-S1.zip › bionlp_uima_repository-1.0.1/docs/api/src-html/edu/uchsc/ccp/util/nlp/parser/corpus/IProLinkCorpus2Parser.html]

```
001    package edu.uchsc.ccp.util.nlp.parser.corpus;
002    
003    import java.io.BufferedReader;
004    import java.io.IOException;
005    import java.util.ArrayList;
006    import java.util.List;
007    import java.util.regex.Matcher;
008    import java.util.regex.Pattern;
009    
010    import edu.uchsc.ccp.util.nlp.GenericTextUtils;
011    import edu.uchsc.ccp.util.nlp.annotation.AnnotationSet;
012    import edu.uchsc.ccp.util.nlp.annotation.Annotator;
013    import edu.uchsc.ccp.util.nlp.annotation.TextAnnotation;
014    import edu.uchsc.ccp.util.nlp.document.GenericDocument;
015    import edu.uchsc.ccp.util.nlp.ei.xml.XMLEntityExtractor;
016    import edu.uchsc.ccp.util.nlp.mention.ClassMentionTypes;
017    
018    /**
019     * A parser for the iProLink corpus2.xml file.  This class outputs the original text and accompanying annotations for each corpus document. Titles and abstracts
020     * are outputted as separate documents.<br>
021     * <br>
022     * Output Classes:<br>
023     * <ul>
024     * <li>protein</li>
025     * <li>long-form proteins</li>
026     * </ul>
027     * @author williamb
028     *
029     */
030    public class IProLinkCorpus2Parser extends CorpusParser {
031        public static final int DOCUMENT_COLLECTION_ID = 9;
032        
033        private static final String PROTEIN_TAG = "protein";
034        private static final String LONG_FORM_PROTEIN_TAG = "long_form";
035        
036        private GenericDocument nextTitleDocument = null;
037        private GenericDocument nextAbstractDocument = null;
038        private boolean returnedTitleDocument = false;
039        private boolean returnedAbstractDocument = false;
040        
041        private BufferedReader br;
042        
043        private final Annotator annotator =  new Annotator(new Integer(11),"","iProLink","PIR");
044        private final AnnotationSet annotationSet = new AnnotationSet(new Integer(9),"iProLink prot/long Gold Annotations","<protein>, <long_form> annotations extracted from the iProLink corpus2.xml file.");
045        
046        @Override
047        public void close() throws IOException {
048        }
049    
050        @Override
051        public boolean hasNext() {
052            /* if we have not yet returned the abstract document, then return true */
053            if (returnedTitleDocument && !returnedAbstractDocument) {
054                return true;
055            } else {
056                /* we have already returned the abstract document, so check to see if there are new title and abstract documents to return */
057                try {
058                    return hasNextDocument();
059                } catch (IOException e) {
060                    error("Error while parsing the IProLink Corpus 1 file.");
061                    e.printStackTrace();
062                    return false;
063                }    
064            }
065        }
066    
067        @Override
068        public GenericDocument next() {
069            if (!returnedTitleDocument) {
070                returnedTitleDocument = true;
071                return nextTitleDocument;
072            } else {
073                returnedAbstractDocument = true;
074                return nextAbstractDocument;
075            }
076        }
077        
078        public IProLinkCorpus2Parser(String corpus2XMLFile) throws IOException {
079            br = GenericTextUtils.openReader(corpus2XMLFile);
080        }
081        
082        
083        /**
084         * corpus1.xml has tags: protein, acronym, and compound-protein
085         * @param iProLinkCorpus1File
086         * @param connectionPropertiesFile
087         * @throws IOException
088         */
089        public boolean hasNextDocument() throws IOException {
090            returnedAbstractDocument = false;
091            returnedTitleDocument = false;
092            String line;
093    
094            Pattern idPattern = Pattern.compile("PMID:(\\d+)");
095            Pattern titlePattern = Pattern.compile("TI\\s+-\\s?(.*)");
096            Pattern abstractPattern = Pattern.compile("AB\\s+-\\s?(.*)");
097            Matcher m;
098    
099            String titleText = null;
100            String abstractText = null;
101            String id = null;
102            boolean inAbstract = false;
103            boolean inTitle = false;
104    
105            while ((line = br.readLine()) != null) {
106                if (line.startsWith("PMID")) {
107                    m = idPattern.matcher(line);
108                    if (m.find()) {
109                        id = m.group(1);
110                    } else {
111                        System.err.println("WARNING -- IProLink: ID expected but not found.");
112                    }
113                } else if (line.startsWith("TI  - ")) {
114                    m = titlePattern.matcher(line);
115                    if (m.find()) {
116                        titleText = m.group(1);
117                        inTitle = true;
118                        inAbstract = false;
119                    } else {
120                        System.err.println("WARNING -- IProLink: Title expected but not found.");
121                    }
122                } else if (line.startsWith("AB  - ")) {
123                    m = abstractPattern.matcher(line);
124                    if (m.find()) {
125                        abstractText = m.group(1);
126                        inAbstract = true;
127                        inTitle = false;
128                    } else {
129                        System.err.println("WARNING -- IProLink: Abstract expected but not found.");
130                    }
131                } else if (line.startsWith("</DOC>")) {
132                    inAbstract = false;
133                    inTitle = false;
134    
135                    System.out.println("ID: " + id);
136                    System.out.println("TITLE: " + titleText);
137                    System.out.println("ABSTRACT: " + abstractText);
138                    
139                    
140                    /* search the titlefor entities */
141                    String documentID = id + "-t";
142                    
143                    String titleWithProteins = titleText.replaceAll("</?" + LONG_FORM_PROTEIN_TAG + ">","");
144                    String titleWithLongForms = titleText.replaceAll("</?" + PROTEIN_TAG + ">","");
145                    
146                    List<TextAnnotation> proteinAnnotations = XMLEntityExtractor.parseEntitiesFromSparseUntokenizedXML(titleWithProteins, PROTEIN_TAG, PROTEIN_TAG, documentID, DOCUMENT_COLLECTION_ID, ClassMentionTypes.PROTEIN, annotator, annotationSet);
147                    List<TextAnnotation> longFormAnnotations = XMLEntityExtractor.parseEntitiesFromSparseUntokenizedXML(titleWithLongForms, LONG_FORM_PROTEIN_TAG, LONG_FORM_PROTEIN_TAG, documentID, DOCUMENT_COLLECTION_ID, ClassMentionTypes.PROTEIN_LONG_FORM, annotator, annotationSet);
148                    
149                    
150                    /* store the untagged title text */
151                    /* remove html tags from the title text */
152                    titleText = titleText.replaceAll(GenericTextUtils.escapeCharacterForRegEx("<" + PROTEIN_TAG + ">"), "").replaceAll(GenericTextUtils.escapeCharacterForRegEx("</" + PROTEIN_TAG + ">"), "");
153                    titleText = titleText.replaceAll(GenericTextUtils.escapeCharacterForRegEx("<" + LONG_FORM_PROTEIN_TAG + ">"), "").replaceAll(GenericTextUtils.escapeCharacterForRegEx("</" + LONG_FORM_PROTEIN_TAG + ">"), "");
154                    
155                    List<TextAnnotation> titleAnnotations = new ArrayList<TextAnnotation>();
156                    titleAnnotations.addAll(proteinAnnotations);
157                    titleAnnotations.addAll(longFormAnnotations);
158                    
159                    /* create two new Generic Documents, one for the title and one for the abstract */
160                    nextTitleDocument = new GenericDocument();
161                    nextTitleDocument.setDocumentText(titleText);
162                    nextTitleDocument.setAnnotations(titleAnnotations);
163                    nextTitleDocument.setDocumentID(id + "-t");
164                    
165                    /* now search the abstract */
166                    documentID = id + "-a";
167                    
168                    String abstractWithProteins = abstractText.replaceAll("</?" + LONG_FORM_PROTEIN_TAG + ">","");
169                    String abstractWithLongForms = abstractText.replaceAll("</?" + PROTEIN_TAG + ">","");
170                    
171                    proteinAnnotations = XMLEntityExtractor.parseEntitiesFromSparseUntokenizedXML(abstractWithProteins, PROTEIN_TAG, PROTEIN_TAG, documentID, DOCUMENT_COLLECTION_ID, ClassMentionTypes.PROTEIN, annotator, annotationSet);
172                    longFormAnnotations = XMLEntityExtractor.parseEntitiesFromSparseUntokenizedXML(abstractWithLongForms, LONG_FORM_PROTEIN_TAG, LONG_FORM_PROTEIN_TAG, documentID, DOCUMENT_COLLECTION_ID, ClassMentionTypes.PROTEIN_LONG_FORM, annotator, annotationSet);
173                    
174                    /* store the untagged abstract text */
175                    /* remove html tags from the abstract text */
176                    abstractText = abstractText.replaceAll(GenericTextUtils.escapeCharacterForRegEx("<" + PROTEIN_TAG + ">"), "").replaceAll(GenericTextUtils.escapeCharacterForRegEx("</" + PROTEIN_TAG + ">"), "");
177                    abstractText = abstractText.replaceAll(GenericTextUtils.escapeCharacterForRegEx("<" + LONG_FORM_PROTEIN_TAG + ">"), "").replaceAll(GenericTextUtils.escapeCharacterForRegEx("</" + LONG_FORM_PROTEIN_TAG + ">"), "");
178                                    
179                    List<TextAnnotation> abstractAnnotations = new ArrayList<TextAnnotation>();
180                    abstractAnnotations.addAll(proteinAnnotations);
181                    abstractAnnotations.addAll(longFormAnnotations);
182                    
183                    nextAbstractDocument = new GenericDocument();
184                    nextAbstractDocument.setDocumentText(abstractText.trim());
185                    nextAbstractDocument.setAnnotations(abstractAnnotations);
186                    nextAbstractDocument.setDocumentID(id + "-a");
187    
188                    return true;
189                    
190                } else {
191                    /* there are line breaks in the abstract, so if we are in the abstract, we add the line to the abstract text */
192                    if (inAbstract) {
193                        abstractText += (" " + line.trim());
194                    } else if (inTitle) {
195                        titleText += (" " + line.trim());
196                    } else {
197                        System.err.println("WARNING -- IProLink: Unexpected tag at beginning of line: " + line);
198                    }
199                }
200            }
201            
202            return false;
203            
204            
205        }
206    
207    }
```
